# Supplementary material for: Mechanistic Insights in NeuroD Potentiation of Mineralocorticoid Receptor Signaling
Source: Int J Mol Sci. 2019 Mar 29;20(7):1575. doi: 10.3390/ijms20071575 (PMC6479562; doi:10.3390/ijms20071575)
Supplement: Supplementary file 1 [file ijms-20-01575-s001.pdf]

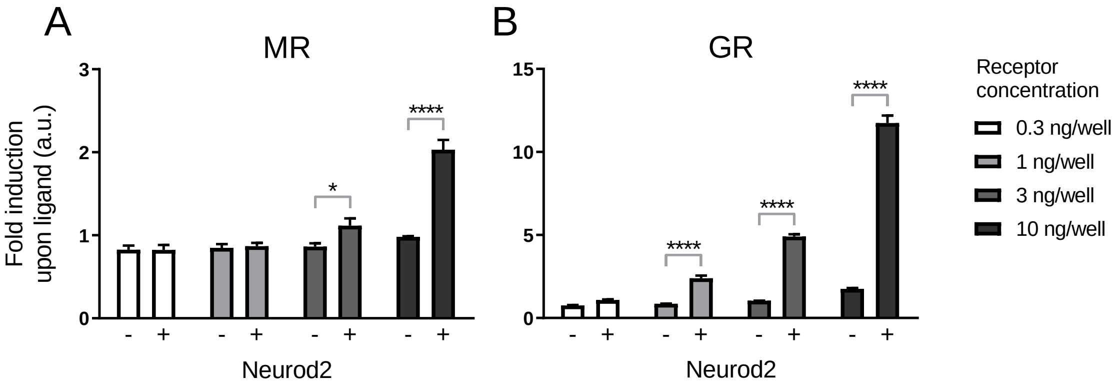

Figure S1. Neurod2 effect as a result of nuclear receptor titration for (**A**) MR and (**B**) GR. HEK293 cells were transfected with GRE-At_GC luciferase construct, and various amounts of MR or GR (0.3-1-3-10 ng/well), with or without Neurod2 (10 ng/well), and stimulated with corticosterone (10^{-7} M). Data are presented as luciferase activity fold induction upon corticosterone treatment. a.u. = arbitrary unit; * P<0.05, **** P<0.0001
